# Recent Advancements in the Synthesis of Functional Polyolefins by Non-Bridged Half-Titanocenes

**DOI:** 10.3390/molecules30010039

**Published:** 2024-12-26

**Authors:** Yanjun Chen, Haiqian Dong

**Affiliations:** 1 Ningbo Key Laboratory of High Performance Petroleum Resin Preparation Engineering and Technology, Ningbo Polytechnic, Ningbo 315800, China; 2 College of Chemical Engineering, Ningbo Polytechnic, Ningbo 315800, China; 2274723205@nbpt.edu.cn

**Keywords:** functional polyolefins, half-titanocene, copolymerization

## Abstract

Polyolefins are used widely due to their benefits such as being lightweight, chemical inertness, low cost, tunable properties, and easy processability. However, their nonpolar nature significantly limits their high-end applications. The non-bridged half-titanocenes exhibit remarkable catalytic activities with good comonomer incorporations in the olefin polymerization. The synthesis of functional polyolefins has attracted more and more attention recently. The non-bridged half-titanocenes have been used in the preparation of functional polyolefins, in particular the functional olefin copolymers. Herein, the recent advancements in the synthesis of functional polyolefins by non-bridged half-titanocenes were reviewed. The functional polyolefins have been synthesized by direct copolymerization of olefin with functional comonomers using half-titanocenes as precatalysts. In addition, polyolefins containing reactive groups could be synthesized by the olefin (co)polymerization using half-titanocenes as precatalysts. The functional polyolefins were synthesized successfully by the post-functionalization of polyolefin containing reactive groups.

## 1. Introduction

Polyolefins are the most widely used polymer materials and constitute over half of global plastic production [1,2,3,4,5]. Although polyolefins have many benefits, such as being lightweight, chemical inertness, low cost, tunable properties, and easy processability, their nonpolar nature significantly limits their high-end applications. The incorporation of polar groups into polyolefin chains provides an efficient way to improve surface properties, leading to the improvement of adhesion, conductivity, dyeability, printability, and compatibility with other polymers and inorganic fillers in the resultant functionalized polyolefins. Therefore, the efficient introduction of polar groups into hydrophobic polyolefins has attracted much attention from both academic and industrial communities [6,7,8,9,10,11,12,13,14,15,16,17,18,19]. The precise and efficient synthesis of polyolefins containing polar groups has been successfully realized by the direct coordination copolymerization of the olefins with polar monomers using a late transition metal catalyst. However, functional polyolefins with certain branching were usually afforded due to the “chain-walking” mechanism [15,16,17,18,19]. In addition, it is still a challenge to prepare precise copolymers of ethylene with α-olefin or styrene for the late transition metal catalysts. By contrast, the early transition metal catalysts exhibit good catalytic behavior in the preparation of linear polyethylene and precise copolymers of ethylene with α-olefin or styrene [20,21,22]. However, both the catalytic activity and incorporation ratio were significantly affected due to the strong interaction of the heteroatom with the active species. Alternatively, the precise functional polyolefins have been successfully afforded by the copolymerization of olefin with protected polar monomer using early transition metal catalysts. Furthermore, the precise functional polyolefins could also be prepared by the post-modification of polyolefins with reactive groups, which were prepared by the copolymerization of olefin with monomers containing reactive groups using early transition metal catalysts [23,24,25]. Catalysts play important roles in the copolymerization of olefin with protected polar monomer and the copolymerization of olefin with monomers containing reactive groups. The non-bridged half-titanocenes exhibit remarkable catalytic activities with good comonomer incorporations in the olefin copolymerizations [26,27,28,29,30], which have also been used in the preparation of precise functional polyolefins. Herein, the recent advancements in the synthesis of functional polyolefins by non-bridged half-titanocenes were reviewed.

## 2. Synthesis of Functional Polyolefins by Direct Copolymerization of Olefin with Functional Comonomers

The direct copolymerization of olefin with functional monomers is one of the effective approaches to prepare functional polyolefins. However, the functional groups in the comonomer greatly affect the active center during the polymerization, leading to transfer or termination [13,17,23,24]. Therefore, only special functional comonomers or the protected comonomers could be used in the synthesis of functional polyolefins by the direct copolymerization of olefin with functional comonomers. Different olefin monomers could be used in the direct copolymerization of olefin with functional comonomers, affording different functional polyolefins, such as functional polyethylene and functional polyolefin elastomer.

### 2.1. Synthesis of Functional Polyethylene

#### 2.1.1. Synthesis of Functional Polyethylene Containing SiR_3_ (R = Me, iPr) Groups

**Figure 1:**
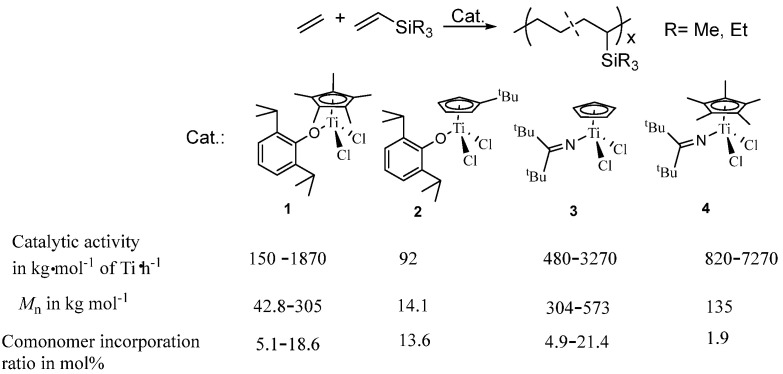
Synthesis of ethylene-ATMS copolymers by using half-titanocenes **1**–**4**.

The alkenylsilanes could be used as functional comonomers for the preparation of functional polyethylene containing SiH_3_ groups. However, the silane group plays a role of chain transfer reagent, leading to functional polyethylenes with long-chain branching and low molecular weight (*M*_n_ = 4.6–78.6 kg·mol^−1^) [31,32]. Compared to SiH_3_, the SiMe_3_ group should be more easily incorporated into the copolymer during the copolymerization of ethylene with alkenylsilanes. The copolymerization of ethylene with allyltrimethylsilane (ATMS) by Cp_2_ZrCl_2_ or [(CH_2_)_2_(indenyl)_2_]ZrCl_2_/MAO catalysts [33] also led to functional polyethylenes with low molecular weight (*M*_w_ = 1.2–2.9 kg mol^−1^), due to the ß-hydrogen elimination after the insertion of bulky ATMS. Due to the unique characteristics of half-titanocenes containing anionic ancillary donor ligands for the copolymerization of ethylene with other olefins, these half-titanocenes were used for the efficient synthesis of functional polyethylene containing SiR_3_ (R = Me, iPr) groups by the copolymerization of ethylene with vinyltrialkylsilanes (VTMS) [34] or allyltrialkylsilane (ATMS) [35]. The catalytic activity of the half-titanocene is higher in the copolymerization of ethylene with ATMS than that in the copolymerization of ethylene with VTMS due to ATMS being less bulky. Both the catalytic activity of the catalyst and the incorporation ratio of ATMS are obviously influenced by the chemical structure of half-titanocenes. As shown in Figure 1, the half-titanocenes **3** and **4** containing ketimide ligands exhibited higher catalytic activities than that of half-titanocenes **1** and **2** containing phenoxide ligands under similar polymerization conditions. In particular, half-titanocene **4** exhibited the highest catalytic activity of 41,600 kg copolymer·mol^−1^ of Ti·h^−1^. The half-titanocenes **1** and **2** exhibited better copolymerization ability than their analogs **3** and **4**. The ethylene-ATMS copolymers with a high ATMS incorporation of 24.4 mol% and 25.9 mol% were afforded by using half-titanocenes **1** and **2** as precatalysts, respectively. Interestingly, the molecular weight (*M*_n_ = 116–370 kg·mol^−1^) of the resulting ethylene-ATMS copolymers prepared by half-titanocenes **3** and **4** was much higher than that (*M*_n_ = 12.7–28.7 kg·mol^−1^) of ethylene-ATMS copolymers prepared by half-titanocenes **1** and **2** [35].

#### 2.1.2. Synthesis of Functional Polyethylene Containing OH Groups

**Figure 2:**
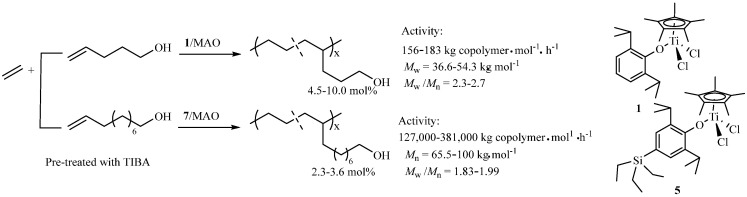
Synthesis of functional polyethylene with OH groups.

Due to the coordination of the lone-pair electrons of heteroatoms in the polar monomer to the metal center of the active site, the low incorporation of the polar monomer was usually observed. In addition, the steric bulkiness also leads to the low incorporation of the polar monomer. The half-titanocenes have been reported as efficient catalysts for ethylene copolymerization with some bulky olefins [26,27,28,29,30]. Therefore, the copolymerization of ethylene with protected 4-penten-1-ol (4P1O) by triisobutylaluminum (TIBA) was investigated by using the half-titanocene catalyst **1**, as shown in Figure 2. The functional polyethylene containing ~10 mol% of OH groups was prepared with relatively low molecular weight (*M*_w_ = 36.6 kg/mol) [36].

**Figure 3:**
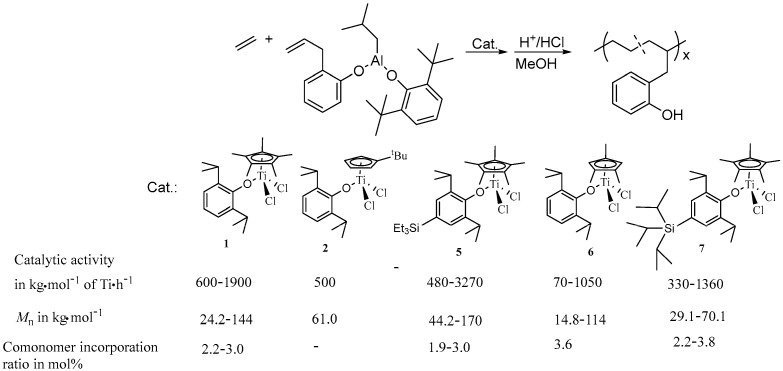
Synthesis of functional polyethylenes by the copolymerization of ethylene with 2-allylphenol.

The introduction of SiMe_3_ and SiEt_3_ groups to the para-position of phenoxide ligands in half-titanocenes leads to a remarkable improvement in catalytic activity and copolymerization ability [37]. The copolymerization of ethylene with TIBA protected 9-decen-1-ol (DC-OH) using the half-titanocene containing SiEt_3_ group (**5**, as shown in Figure 2) showed high catalytic activity (381,000 kg copolymer·mol^−1^ of Ti·h^−1^), affording functional polyethylene containing 2.3–3.6 mol% of OH groups with relatively high molecular weight (*M*_n_ = 65.5–100 kg/mol) and unimodal molecular weight distribution (*M*_w_/*M*_n_ = 1.83) [37]. Interestingly, these catalysts can also be used in the copolymerization of ethylene with 2-allylphenol (AP), a biomimetic fungicide that mimics the compound ginkgol found in gingko fruit [38]. Before the copolymerization, the AP comonomer was pretreated with the TIBA to protect the hydroxy group as Al-phenoxide. The half-titanocene **5** exhibited a high catalytic activity of 2430 kg copolymer·mol^−1^ of Ti·h^−1^. The copolymerization using **5** gave functional polyethylenes with high molecular weight (*M*_n_ = 13.2–113 kg/mol), relatively low *M*_w_/*M*_n_ values (*M*_w_/*M*_n_ = 1.70–2.30) and uniform compositions at relatively low AP concentration. A new approach for the protection of the hydroxy group in the AP comonomer by using TIBA and 2,6-*^t^*Bu_2_-C_6_H_3_OH was proposed, as shown in Figure 3. The catalytic activity of **5** is higher than that of its analogs, as shown in Figure 3. In addition, the catalytic activity increased to 3270 kg copolymer·mol^−1^ of Ti·h^−1^ due to the new protection approach. The functional polyethylenes with higher contents of AP units (1.9–3.8 mol%) and molecular weight (*M*_n_ = 44.2–170 kg/mol) were afforded using the **5**/MAO catalytic system [38].

### 2.2. Synthesis of Functional Polyolefin Elastomer

**Figure 4:**
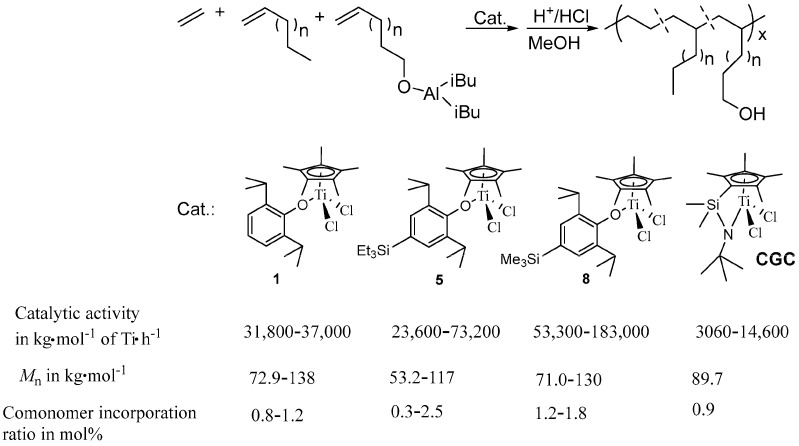
Synthesis of functional polyolefin elastomers by the copolymerization of ethylene with 1-dodecene and 9-decen-1-ol.

Polyolefin elastomer (POE) is a random copolymer of ethylene with α-olefin (with a high content of over 20 wt%) prepared by the coordination copolymerization of ethylene with 1-octene, 1 hexene, or 1-butene [39]. The high-performance POE exhibits excellent mechanical and elastomeric properties, which enables it to attract much attention in the industry. Similarly to the polyethylene, the lack of polar groups in the macromolecular chain limits its wide application. The half-titanocenes have been used as efficient catalysts for the preparation of POE [26,27,28,29,30]. However, the reports for the preparation of functional POE by the direct copolymerization using half-titanocene catalysts is limited. Nomura et al. reported that the half-titanocene/MAO catalytic system can efficiently catalyze the copolymerization of ethylene with long chain 1-dodecene (DD) and polar monomer 9-decen-1-ol (DC-OH), as shown in Figure 4 [37]. Compared to the catalytic activity of the CGC catalyst (14,600 kg copolymer·mol^−1^ of Ti·h^−1^), the catalytic activity of half-titanocenes **1**, **5**, and **8** is much higher (37,000~638,000 kg copolymer·mol^−1^ of Ti·h^−1^) in the copolymerization of ethylene with DD and DD-OH. In particular, the incorporation ratio of DD-OH (1.2–1.3 mol%) in the copolymerization catalyzed by **5** and **8** is higher than that (0.9%) catalyzed by the CGC catalyst. Furthermore, the functional POE with OH groups (0.8 mol%) and high molecular weight (*M*_n_ = 138 kg·mol^−1^), narrow molecular distribution (*M*_w_/*M*_n_ = 1.83), and a high DD incorporation ratio (15.2%) was afforded by using the advanced half-titanocene catalyst [38].

**Figure 5:**
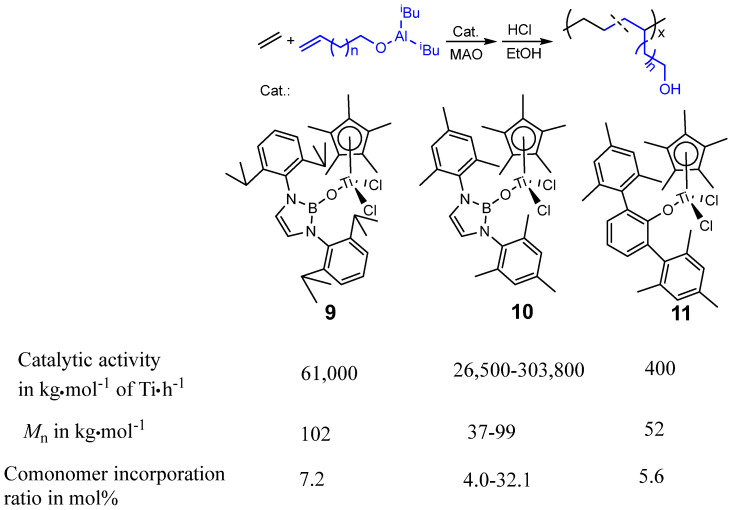
Synthesis of functional polyolefin elastomers by the copolymerization of ethylene with long chain α-olefins containing OH group.

The half-titanocenes supported by Cp* and the boryloxy ligands have been shown to efficiently catalyze the copolymerization of ethylene with 9-decen-1-ol, affording poly(ethylene-co-9-decen-1-ol) with high catalytic and copolymerization ability, as shown in Figure 5 [40]. In the same polymerization conditions, the catalytic activity of precatalyst **10** (2.99 × 10^8^ g·mol^−1^ of Ti·h^−1^) and **9** (6.10 × 10^7^ g·mol^−1^ of Ti·h^−1^) is much higher than that of **7** (4.0 × 10^5^ g·mol^−1^ of Ti·h^−1^). In addition, both the molecular weight and the polar comonomer contents (I) of resulting copolymers prepared by using **9** (*M*_n_ = 102 kg/mol, I = 7.2 mol%) and **10** (*M*_n_ = 90 kg/mol, I = 10.8 mol%) are higher than those of resulting copolymers prepared by 11 (*M*_n_ = 52 kg/mol, I = 5.6 mol%). More importantly, the functional polyolefin elastomers with high polar comonomer contents of up to 32.1 mol% were afforded by using **10** as a precatalyst. All the results indicate that the catalytic activity, copolymerization ability, and molecular weight of the resulting copolymer could be obviously improved by introducing boryloxy ligand. Moreover, the resulting hydroxyl-functionalized copolymers exhibit high strength and toughness (high stress at break of up to 33.9 MPa and a high strain value of 670%) due to the hydrogen bonds in the polymer network [40].

## 3. Synthesis of Functional Polyolefins by Post-Functionalization of Polyolefin Containing Reactive Groups

Although the direct copolymerization of olefin with functional monomers is effective for the preparation of functional polyolefins, it is still a challenge to prepare functional polyolefins with both high molecular weight and high polar monomer incorporation by the direct copolymerization approach. The post-functionalization of polyolefin containing reactive groups is another approach to prepare functional polyolefins [23,24]. In this approach, the polyolefin containing reactive groups is prepared first by the copolymerization of olefin with another olefin monomer containing reactive groups (for example: C=C double bond). Then, the reactive groups are transferred to functional groups by some reactions such as an oxidization reaction and a thio-ene click reaction. In addition, functional polyolefins containing both a high content of polar groups and a high molecular weight could be afforded. More importantly, various functional groups including hydroxyl, epoxy, and carboxylic groups have been introduced into the polyolefin macromolecular chain [23,24]. The half-titanocenes display high catalytic activity and copolymerization ability for the polymerization and copolymerization of various olefins. In particular, polyolefins with a high content of olefin units containing reactive groups could be successfully prepared using half-titanocene catalysts [41,42,43,44,45]. Herein, the preparation of functional polyolefins by the post-functionalization of polyolefin containing reactive groups, which have been prepared using half-titanocene catalysts, was reviewed.

### 3.1. Synthesis of Functional Polyethylene by Post-Functionalization Method

**Figure 6:**
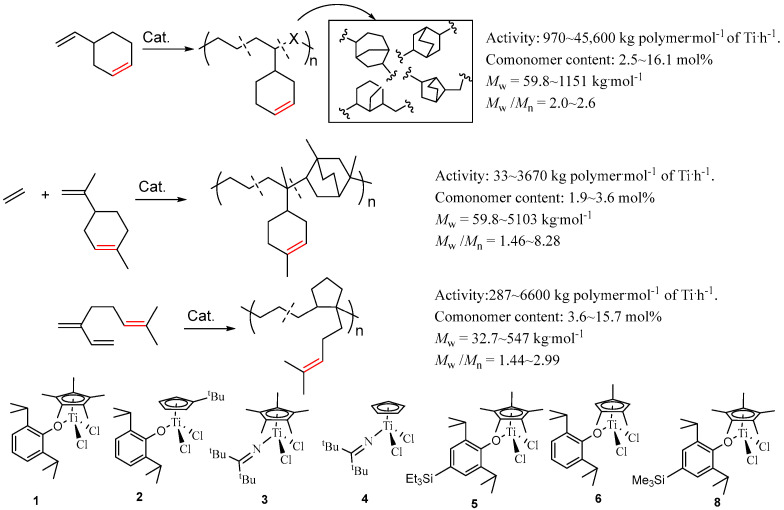
Synthesis of polyethylenes containing pendent olefin groups in the side chain.

The pendent olefin groups of polyolefins can be converted to polar functional groups through chemical modifications under mild conditions. Therefore, the synthesis of polyolefins with olefinic double bonds in the pendent side chains is critical for the preparation of functional polyethylene by the post-functionalization method. Usually, the dienes were used as the comonomer to copolymerize with ethylene to introduce the olefinic double bonds in the pendent side chains without cross-linking. The copolymerization of ethylene with dienes containing two olefins with different reactivities afforded copolymers with pendent olefinic double bonds. The half-titanocenes exhibit excellent catalytic behavior toward the copolymerization of ethylene with various dienes [41,42,43,44,45]. As shown in Figure 6, the half-titanocenes containing anionic oxygen or nitrogen ligands exhibit moderate to high catalytic activity (97–45,600 kg polymer·mol^−1^ of Ti·h^−1^) for the copolymerization of ethylene with vinylcyclohexene (VCH), affording copolymers with low to high molecular weight (*M*_w_ = 59.8–1151 kg·mol^−1^), narrow molecular weight distribution (*M*_w_/*M*_n_ = 2.0~2.6), and a high VCH incorporation ratio of up to 16.1 mol% [43]. The half-titanocene **4** containing ketimide ligand and Cp ligand showed higher catalytic activity than that of half-titanocene **1** containing phenoxide and Cp* ligands as well as half-titanocene **3** containing ketimide and Cp* ligands [41]. The copolymer with a high VCH incorporation ratio provides starting materials for the preparation of functional ethylene with high polar group content [41].

Limonene, which is an abundant natural product, can be considered a promising monomer. Compared to VCH, limonene contains one 1,1-disubstituted α-olefin structure and a trisubstituted cycloolefin structure. Although limonene can be used to prepare polyolefin containing pendent olefinic double bonds, the coordination polymerization of limonene is still a challenge [42,43]. The half-titanocenes show good catalytic activity toward the copolymerization of ethylene with limonene, affording ethylene copolymers with pendent olefinic double bonds. In particular, the ethylene-limonene copolymers with high molecular weight (*M*_w_ up to 128 kg·mol^−1^), uniform molecular weight distribution (*M*_w_/*M*_n_ = 1.37–3.19), and a good limonene incorporation ratio (1.9~3.6 mol%) could be prepared by using the half-titanocene **6**/MAO catalytic system with catalytic activity in the range of 55–714 kg polymer·mol^−1^ of Ti·h^−1^ [43].

β-myrcene (My) containing three double bonds (one conjugated 1,3-diene framework and one trisubstituted double bond) is also a good candidate for the preparation of polyoefin containing pendent olefinic double bonds. However, the coordination copolymerization of ethylene with β-myrcene is still a big challenge. The report of this copolymerization is limited [44,45]. As shown in Figure 6, the half-titanocene catalysts showed rather efficient My incorporation in the copolymerization of ethylene with My. The half-titanocenes containing phenoxide ligand **1**, **2**, and **5** showed higher catalytic activity than their analog **4** containing ketimide ligand. The catalytic activity of half-titanocene **5** for ethylene/My copolymerization could be up to 6600 kg polymer·mol^−1^ of Ti·h^−1^, which is slightly higher than that (6100 kg polymer·mol^−1^ of Ti·h^−1^) of half-titanocene **1**. The ethylene/My copolymers with moderate to high molecular weight (*M*_w_ = 32.7–547 kg·mol^−1^), unimodal molecular weight distributions, and uniform composition as well as a high My incorporation ratio (up to 15.7 mol%) were afforded [45].

**Figure 7:**
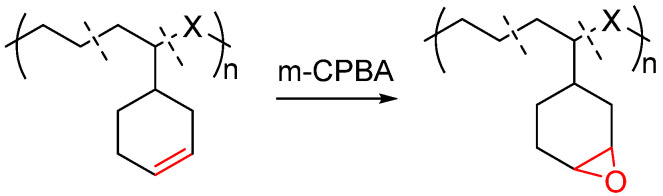
Synthesis of polyethylene containing epoxy functional groups.

The polyolefin containing pendent olefinic double bonds could be easily functionalized by the reaction of C=C double bonds. As shown in Figure 7, the epoxidation of C=C double bonds in the ethylene/VCH copolymer was successfully realized by using m-chloroperbenzoic acid (m-CPBA) as oxidation reagent in CHCl_3_ at room temperature. The NMR characterization of the resulting functional polyethylene demonstrates a facile functionalization of the olefinic double bond and an introduction of epoxy functional groups have been achieved quantitatively [41].

### 3.2. Synthesis of Functional Poly(α-Olefin) by Post-Functionalization Method

**Figure 8:**
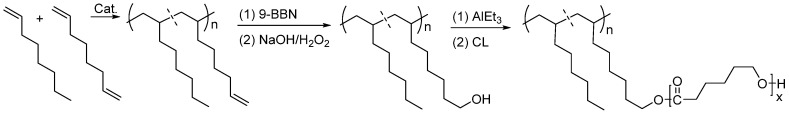
Synthesis of poly(1-octene) with OH functional groups or PCL side chain.

The half-titanocenes have also been used as catalysts for the polymerization of α-olefin. In addition, the polymerization of 1,7-octadiene catalyzed by half-titanocene **1** and the **3**/MAO catalytic system afforded polymers with terminal olefinic double bonds in the side chain. Therefore, the copolymerization of 1,7-octadiene and 1-octene was carried to prepare copolymers with double bonds in the side chain. The catalytic activity of the **1**/MAO catalytic system for the copolymerization is as high as 8010 kg polymer·mol^−1^ of Ti·h^−1^, leading to copolymers with high molecular weight (*M*_w_ = 660–991 kg·mol^−1^) and uniform molecular weight distribution (*M*_w_/*M*_n_ = 1.52–1.77). In addition, the content of the double bonds in the copolymers can be controlled in the range of 1.6–37.3 mol% by the feeding ratio of 1,7-octadiene with 1-octene. As shown in Figure 8, the double bonds in the side chain of the copolymers have been converted to -CH_2_-CH_2_-OH functional groups by treating the copolymers with 9-BBN and then NaOH/H_2_O_2_. Furthermore, the OH groups were used as initiating sites to initiate the ring opening polymerization of ε-caprolactone(CL), affording graft copolymers of poly(1-octene)-graft-poly(CL) by the “grafting from” method. The molecular weight of the grafting poly(CL) can be controlled by the polymerization time of CL [45].

### 3.3. Synthesis of Functional Ethylene-Propylene Elastomer by Post-Functionalization Method

**Figure 9:**
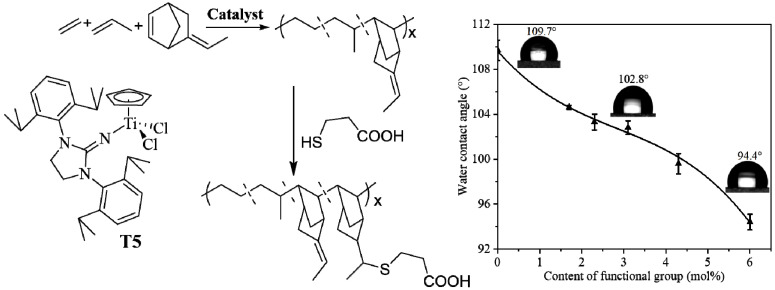
Synthesis and properties of functional EPDM.

Ethylene-propylene elastomers, for example, ethylene-propylene-diene rubber (EPDM), have been widely used as sealing materials, wires, and cables. This kind of elastomer exhibits superior elasticity and flexibility at low temperature, excellent heat resistance, and good electrical insulation property. However, the nonpolar nature of the polymer chain significantly limits its high-end applications. The introduction of polar functional groups into the polymer chain could dramatically improve the properties of ethylene-propylene elastomer such as adhesion, dyeability, printability, and compatibility with polar polymers and inorganic fillers [46,47]. The commercial EPDM is a terpolymer of ethylene, propylene, and non-conjugated diene, which contains C=C double bonds in the side chain [48,49,50]. The double bonds are usually used as cross-linking sites during the vulcanization. From another side, the EPDM could also be considered as “reactive polyolefin intermediate”, in which the C=C double bonds could be modified to obtain functionalized ethylene-propylene elastomer. Nevertheless, the content of ENB in commercial EPDM is usually less than 12 wt%, which limits the amount of polar group content in the resulting functional ethylene-propylene elastomer [51]. The half-titanocenes displayed excellent catalytic behavior toward ethylene/propylene/5-ethylidene-2-norbornene (ENB) or 5-vinyl-2-norbornene (VNB) copolymerization [51,52,53,54]. It has been reported that the efficient terpolymerization of ethylene, propylene, and ENB was successfully afforded using half-titanocene containing the imidazolidin-2-iminato ligand. Both a high ENB conversion of 98% and a high content of ENB units up to 6.8 mol% (20.8 wt%) in the resulting EPDM were observed due to the excellent copolymerization ability of the catalytic system. Through the thiol-ene click reaction with mercaptopropionic acid, the C=C double bonds in the ENB units of the EPDM could be successfully reacted and introduced with carboxylic acid groups. The content of carboxylic acid groups in the functional ethylene-propylene elastomers could be adjusted by the content of the ENB units and the amount of click reagent. The enhanced hydrophilicity was observed in the functional ethylene-propylene elastomers, evidenced by a decreased water contact angle, as shown in Figure 9 [51].

## 4. Conclusions

The synthesis of functional polyolefins using half-titanocenes was reviewed. The half-titanocenes exhibit high catalytic activity and copolymerization ability for the copolymerization of ethylene with other olefin monomers. The functional polyolefins containing SiR3 or OH groups were synthesized by the copolymerization of ethylene with vinyltrialkylsilanes or olefins containing OH groups. The half-titanocenes showed advantages in catalytic activity and polar monomer incorporation. The functional polyolefins with high molar weight and high polar monomer incorporation were afforded. However, the polar group in the functional polyolefin prepared by the direct copolymerization method was limited. The polyolefins containing reactive groups (especial C=C double bonds) were successfully synthesized. The post-functionalization of the polyolefins containing reactive groups resulted in functional polyolefins with epoxy, COOH, and other polar groups. Various functional polyolefins such as functional polyethylenes, functional polyolefin elastomers, functional poly(α-olefins), and functional ethylene-propylene rubbers could be synthesized by the direct copolymerization method or the post-functionalization method. We personally believe that the half-metallocene will attract more and more attention in the synthesis of advanced polyolefin materials, in particular functional polyolefin materials.
